# Boosting Gene Translation by a Short ORF Encoding for a “Nonsense” Peptide Positioned Immediately Upstream

**DOI:** 10.1021/acssynbio.5c00505

**Published:** 2025-10-27

**Authors:** Junyi Cao, Sarah Goldberg, Roee Amit

**Affiliations:** † Department of Biotechnology and Food Engineering, Technion—Israel Institute of Technology, Haifa 32000, Israel; ‡ The Russell Berrie Nanotechnology Institute, Technion—Israel Institute of Technology, Haifa 32000, Israel

**Keywords:** expression boost, nonfunctional open reading frame, target open reading frame, ribosomal stretching, translational reinitiation, translational coupling

## Abstract

We demonstrate a new synthetic genetic component for boosting translational levels in bacteria. Boosting is facilitated by a short and nonfunctional open reading frame (nfORF) that encodes a “nonsense” peptide located upstream of the target gene. The nfORF may be either in-frame or frame-shifted relative to the target open reading frame (tORF), and it contains an internal strong RBS that is immediately adjacent to its stop codon. We characterized 50 nfORFs, which allowed us to build a predictive model for the nfORF sequence, length, and distance from the tORF. Finally, we validated the model by testing three additional unseen nfORFs and demonstrated that the model also accurately predicts a translational boost for another target gene. Our results show how ribosome recruitment can be engineered to boost translation by as much as ×20 by precise engineering of an upstream nfORF. Our finding can be used for a variety of gene expression applications, by increasing expression for potentially any target gene that is produced in *Escherichia coli*.

## Graphical abstract

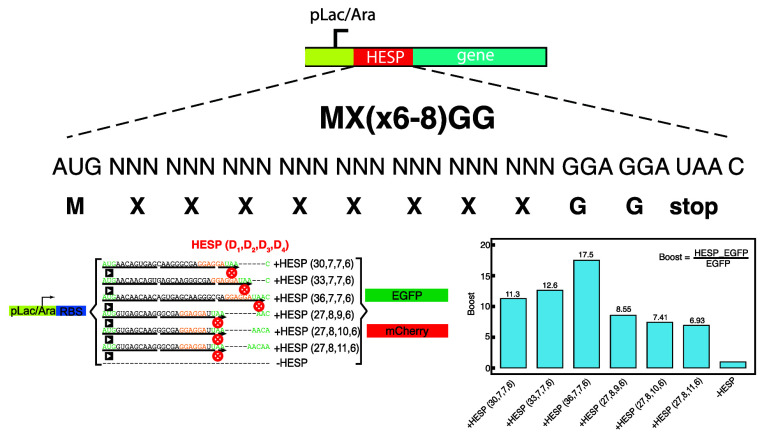

## Introduction

In synthetic biology, the ability to fine-tune protein expression is essential for constructing robust, dynamic gene circuits.^1,2^ Although components of transcriptional control, such as promoters and transcription factors, have long been a main target of genetic engineering, researchers have increasingly recognized the importance of manipulating gene expression at the translational level. Compared to transcriptional regulation, controlling translation offers unique advantages: it enables faster and potentially reversible responses without altering mRNA abundance, and it can exploit diverse RNA-based mechanisms that are often modular and programmable.^3^ As such, an expanding repertoire of translational regulatory tools has emerged, facilitating precision protein output in bacteria, yeast, and even mammalian systems.^4^

Of the plethora of natural and synthetic translational regulatory mechanisms^5–10^ in bacteria, the potential for programmability of two of the simplest mechanisms, translational reinitiation and translational coupling, has been underexplored. Translational reinitiation typically refers to a situation in which the ribosome (usually the 30S subunit) remains bound to the mRNA, rather than fully dissociating after translating the upstream ORF and encountering its stop codon. This partial retention of the ribosomal machinery enables it to bind to the downstream start codon and initiate a subsequent round of translation.^11,12^ While this mechanism can increase the efficiency of producing multiple gene products from a single transcript, it frequently necessitates that upstream translation events terminate at close proximity to the downstream gene to facilitate rapid recycling of some initiation factors and/or ribosomal subunits.^13^ By contrast, translational coupling is a phenomenon in which efficient translation of a downstream ORF depends on the translation of an upstream ORF, but not necessarily by ribosomes “waiting” in place. In many bacterial operons, ribosomes translating upstream genes physically unwind secondary structures or expose downstream ribosome binding sites (RBSs), thereby “coupling” downstream initiation to ongoing upstream translation.^14,15^

To attempt to quantify the regulatory output generated by translational reinitiation and coupling, Tian and Salis developed a predictive biophysical model showing how RBS strength and spacers can be manipulated to coordinate expression levels of neighboring genes within a synthetic operon.^15^ Similarly, another study used fluorescent reporters and RBS modulation to quantify translational coupling in *Escherichia coli* (*E. coli*), revealing that ribosome pausing, spacing, and mRNA folding, can exert complex control on downstream protein production.^14^ These studies revealed that translational coupling can be context-dependent and thus programmable. Namely, if upstream genes are not properly translated or initiation elements remain masked due to structure, downstream initiation may be severely attenuated.

In this work, we build on these earlier studies and explore the programmability aspect of translational reinitiation and coupling by introducing short nonfunctional coding sequences that are positioned upstream of target reporter genes. We demonstrate using a simple regression model that it is possible to predict with a high degree of confidence the translational output of a reporter gene based on the short nonfunctional upstream ORF sequence. Consequently, we demonstrate that ribosome recruitment can be utilized to boost expression of a target gene, by precisely engineering a short nonfunctional ORF (nfORF) upstream to the gene of interest.

## Materials and Methods

### Construction of HESP-Variant Plasmids

The DNA encoding for the hyper-expression short peptide (HESP) nfORF variants (available in Supporting Information Table S1) were ordered from either Integrated DNA Technologies or Sigma-Aldrich, as two reverse-complement oligos per variant, with KpnI and ApaLI overhangs. The oligos were annealed as follows: 20 μL containing μg of each oligo in 1× T4 ligase buffer (New England Biolabs, catalog no. B0202S) were heated to 95 °C for 5 min and then left to cool on the bench for 1 h. Annealed oligos were cloned into a pKan-pLac/Ara-mCherry vector containing a kanamycin resistance gene and an intact mCherry reporter gene, which was linearized using KpnI and ApaLI (New England Biolabs, catalog nos. R3142S and R0507S, respectively), with restriction sites located in the 5′ UTR and within the intact mCherry ORF (i.e., at an internal site upstream of the M1 variant AUG), respectively. The relevant HESP variant annealed band was excised from 1% agarose gel and the column purified (Promega, catalog no. A9281) following restriction to obtain the relevant DNA insertion segment. Ligation was performed using standard T4 ligase protocol (New England Biolabs, catalog no. M0202S). Ligated plasmids were transformed into *E. coli* TOP10 (Invitrogen, catalog no. C404010).

Plasmids for all EGFP variants were constructed as follows: the pKan-pLac/Ara vector was digested with KpnI and XbaI, flanking the mCherry and variable regions, and purified from the gel as described previously. Primers were designed and ordered to amplify the EGFP gene with the relevant upstream sequences from an existing plasmid in the lab via PCR. The resulting PCR products were column purified and subjected to KpnI and XbaI digestion, followed by additional column purification. Digested vector and inserts were ligated using the standard T4 DNA ligase protocol and transformed into *E. coli* TOP10. All plasmids were amplified after overnight growth of harboring *E. coli* strains in LB-Kan ((LB) 10 g of bacto-tryptone, 10 g of NaCl, and 5 g of yeast extract in 1 L of deionized [DI] water, autoclaved; (LB-Kan) LB, supplemented with 25 μg/mL kanamycin). All plasmids were verified using Sanger sequencing.

### Flow Cytometry Measurements

Flow cytometry measurements were performed using a MACSQuant VYB flow cytometer (Miltenyi Biotec), with 561 nm excitation laser and the Y2 detector channel (a 615/20 nm filter) for mCherry and 488 nm excitation laser and detector channel B1 (525/50 nm) for EGFP. The flow cytometer was calibrated using MACSQuant calibration beads (Miltenyi Biotec, catalog no. 130093607) before measurement. Running buffer, washing solution, and storage solution were all purchased from the manufacturer (Miltenyi Biotec, catalog nos. 130092747, 130092749, and 130092748, respectively).

For flow cytometry, *E. coli* TOP10 cells containing the HESP variants were grown overnight in LB-Kan, at 37 °C with 250 rpm shaking. In the morning, 500 μL of bacterial culture was collected and centrifuged at 5000 rpm for 1 min. The pellet was resuspended in 1 mL of 3:1 PBS:LB (PBS, Dulbecco’s phosphate buffered saline, Sartorius, catalog no. 02-023-1A). Samples and appropriate controls were loaded onto a 96-well plate (Thermo Fisher Scientific, catalog no. 167008) in technical duplicates for each clone, each well containing 200 μL of diluted bacterial cells. We note that *E. coli* TOP10 does not require isopropyl ß-d-1-thiogalactopyranoside (IPTG) to express from the Lac/Ara promoter (see Invitrogen guidelines for C404010).

### RNA Extraction and cDNA Sequencing via Nanopore

Starters of *E. coli* TOP10 containing M1 and M1 + 4 plasmids were grown in 5 mL of LB-Kan overnight, at 37 °C with 250 rpm shaking. The following morning, samples were diluted 1:100 in 5 mL of fresh LB-Kan and grown to OD 600 of approximately 0.6. 1.5 mL of culture was pelleted by centrifugation at 6000*g* for 5 min at 4 °C in microcentrifuge tubes. Cell pellets were resuspended in 200 μL of Max Bacterial Enhancement Reagent (Invitrogen, catalog no. 16122012), heated to 95 °C, and incubated for 4 min, after which 1 mL of Trizol (Invitrogen, catalog no. 15596026) was added and mixed via inversion. Samples were then incubated at room temperature for 5 min. For phase separation, 0.2 mL of cold chloroform was added to each tube, and samples were incubated for 3 min, followed by centrifugation at 12000*g* for 15 min at 4 °C. Approximately 600 μL of the upper colorless phase was transferred to a fresh microcentrifuge tube. RNA was precipitated as follows: 0.5 mL of cold isopropanol was added, and samples were incubated for 10 min at room temperature, followed by centrifigation at 15000*g* for 10 min at 4 °C. Supernatant was carefully removed without disturbing the pellet. Pellets were washed via resuspension in 3:1 ethanol:DI water via vortex followed by centrifugation at 7500*g* for 5 min at 4 °C. Supernatant was removed and pellets were allowed to air-dry before final resuspension in 50 μL of RNA-free water (Biolab, catalog no. 23217731, or similar).

Extracted RNA was treated with DNase to remove residual genomic and plasmid DNA using the TURBO DNA-free kit (Invitrogen, catalog no. AM1907). Reverse transcription was done using High-Capacity cDNA Reverse Transcription Kit (Applied Biosystems, catalog no. 4374966), in accordance with kit manufacturer instructions. To enrich for cDNA from the reporter gene and to barcode the samples, we amplified the mCherry reporter region separately for each cDNA sample using the following forward primers for M1 and M1 + 4, respectively, with the underlined sequences acting as barcodes: 5′-CGTTAGGCACACAGAATTCTTAAAGAGGAGAAAGG-3′ and 5′-CGTACTGCACACAGAATTCTTAAAGAGGAGAAAGG-3′. The same reverse primer 5′-GTAGGCCTTGGAGCCGTACA-3′ was used for both samples. The barcoded amplicons were column-purified. The barcoded amplicons were then quantified using a Qubit dsDNA HS Assay (Thermo Fisher Scientific) and pooled at a 1:1 molar ratio for M1 and M1 + 4 samples. The pooled library was purified with 1.0× AMPure XP beads (Beckman Coulter) and eluted in 10 μL of elution buffer (Oxford Nanopore Technologies, SQK-NBD114.24, component EB). The MinION flow cell (FLO-MIN106, Oxford Nanopore Technologies) was primed according to the manufacturer’s instructions, and the library was loaded using 12 μL of DNA library mixed with 2 μL of loading beads and 35 μL of sequencing buffer, following the standard ligation sequencing kit protocol (Oxford Nanopore Technologies, SQK-NBD114.24).

Base calling was performed using Dorado software (Oxford Nanopore Technologies) with the short read setting enabled. After the resulting reads were converted from Fast5 to FastQ file format, read data were processed in three steps, using Python: First, size exclusion was applied, retaining only reads with length within three standard deviations of the mean length. Second, we randomly selected 10 short sequences of 5 bases in length from the expected sequences, and only reads containing at least 8 of 10 sequences were retained. Third, the remaining reads were classified into M1 and M1 + 4 categories based on their respective barcodes. Following the processing, we counted how many of the classified reads matched the designed DNA sequence.

### Protein Extraction and Purification

*E. coli* TOP10 cells harboring the M1 and M1 + 4 plasmids were grown in 5 mL of LB-Kan, at 37 °C and 250 rpm overnight. The following day, the starter content was transferred to 500 mL of TB (24 g of yeast extract, 20 g of tryptone, 4 mL of glycerol, in 900 mL of DI water, autoclaved, and after which 100 mL of 10× phosphate buffer was added; 23.14 g of KH_2_PO_4_, 125.41 g of K_2_HPO_4_, in 1 L of DI water and autoclaved) supplemented with 25 μg/mL kanamycin, and grown overnight at 37 °C and 250 rpm. The next day, cells were allowed to grow until reaching an optical density of >4.5, before being centrifuged at 6000 rpm for 10 min. Supernatant was removed, and the cell pellet was resuspended in 30 mL of resuspension buffer (50 mM Tris base, pH = 8, 100 mM NaCl, 0.02% sodium azide, in 1 L of DI water, titrated to pH = 7), before being passed four times through an EmulsiFlex-C3 homogenizer (Avestin Inc.). Finally, cellular debris was centrifuged at 13000 rpm for 30 min at 4 °C and approximately 35 mL of supernatant was collected.

For each sample, 1 mL of vortexed HisLink Protein Purification Resin (Promega, catalog no. V8821) was transferred to a clean 50 mL centrifuge tube. After allowing the beads to settle, supernatant was removed and beads were resuspended in 2.5 mL of binding buffer (50 mM NaH_2_PO_4_, 300 mM NaCl, 10 mM imidazole, in DI water, adjusted to pH 8.0). Beads were allowed to settle, and 2 mL of binding buffer was removed. Approximately 35 mL of supernatant was added to beads and incubated at room temperature for 1–2 h with headover mixing to allow binding of the His-tagged protein. The beads were washed three times with 5 mL of washing buffer (50 mM NaH_2_PO_4_, 300 mM NaCl, 20 mM imidazole, in DI water, adjusted to pH 8.0) to remove nonspecifically bound proteins. During the third wash, beads were transferred to a gravity column (Biorad, catalog no. 7311550). The target protein was eluted by incubating the beads with 0.5 mL of elution buffer (50 mM NaH_2_PO_4_, 300 mM NaCl, 500 mM imidazole, in DI water, adjusted to pH 8.0) on the column, and the eluates were collected for downstream analysis. Eluates were run on native polyacrylamide gel (10% and 4% resolving and stacking gels, respectively) to verify the mCherry length.

### Liquid Chromatography/Mass Spectrometry

Length-verified eluates were submitted to the Smoler Proteomics Center at the Technion. They were analyzed by LC-MS/MS on HF-X (Thermo), and identified by Discoverer (software version 2.4) with the Sequest search algorithm (Thermo) against the *E. coli* (strain K12) database, a decoy database (in order to determine the false discovery rate), and in addition all possible amino acid sequences of the mCherry ORF with all possible frame shifts. (False discovery rate, noted FDR, is the estimated fraction of false positives in a list of peptides.) All of the identified peptides were filtered with a mass accuracy. High-confidence peptides passed the 1% FDR threshold. Medium-confidence peptides passed the 5% FDR threshold. Semiquantitation was done by calculating the peak area of each peptide. The abundance of the protein is the average of all of the associated peptide group abundances.

## Results

We hypothesized that a short nfORF encoding for a nonsense peptide positioned immediately upstream of a target ORF (tORF) could lead to an increased translation rate of the tORF (Figure 1A). To discover the important characteristics of such an nfORF, we opted to test the effects of various nfORF candidates on the expression of an mCherry encoding tORF. To properly set up the system, we first noticed (Figure 1B) that the mCherry tORF may potentially have up to three in-frame AUG start codons within the first 15 amino acids of the protein that can be used for concomitant and multicistronic translation of mCherry.^16^ We constructed variants M1, M2, and M3, corresponding to the native sequence, the variant lacking the first AUG, and the variant lacking the first two AUGs. We then measured the fluorescence of each mCherry variant. We found (Figure 1C) that M1 generated the strongest fluorescence signal as previously reported,^16^ M2 yielded a non-negligible fluorescent signal, and M3 was nonfluorescent (i.e., completely overlapping the negative control (NC) variant in which a stop codon was inserted within the mCherry gene immediately after the third AUG). Consequently, in order to characterize the regulatory effects of various nfORF variants that encode for hyper-expression short peptides (HESPs), we opted to continue with the M2 variant as the protein product of the tORF.

**Figure 1:**
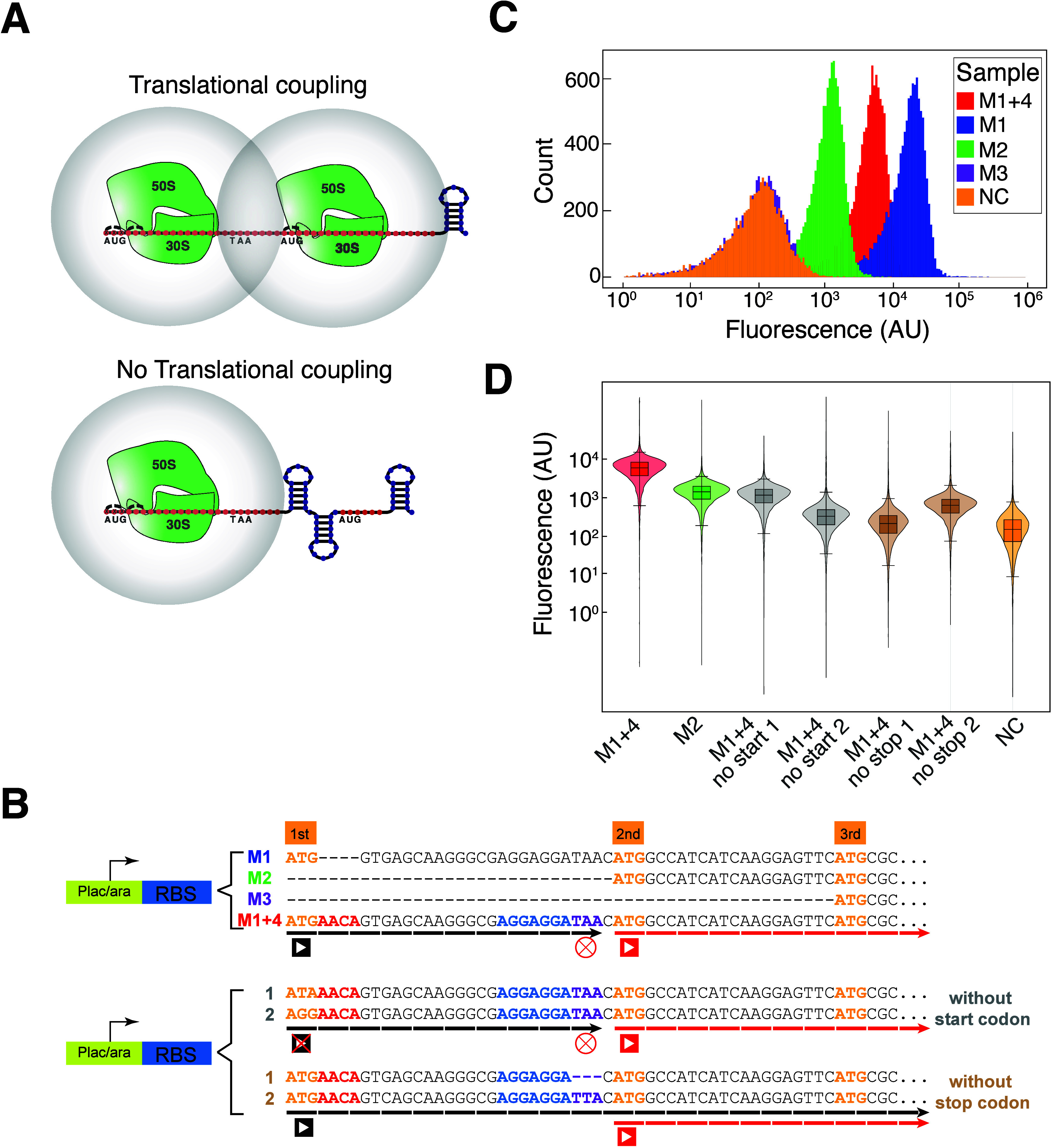
Four nucleotide insertion leading to a boost in truncated mCherry gene fluorescence. (A) Schematic showing a model depiction of translational coupling. (Top) Translation coupling leading to unfolding of RNA and easier access to downstream ORF RBS. (Bottom) With no translational coupling, downstream ORF remaining folded and predominantly inaccessible to ribosome. (B) HESP sequence variants used for the fluorescence measurements depicted in panels C and D. (C) Flow cytometry distributions showing the fluorescence recovery that the M1 + 4 variant achieves over the truncated M2 variant. (D) Flow cytometry distributions depicted as violin plots for all HESP variants whose sequences are depicted in panel B.

To provide a proof-of-concept for the potential regulatory role for such an nfORF via translational coupling, we first inserted a four-base (AACA) quadmer within the M1 sequence. This quadmer introduces a frameshift mutation that effectively converts the first AUG and the ten immediate downstream codons into a separate ORF encoding for a HESP variant, as it positions a UAA stop codon immediately adjacent to and out-of-frame to the second AUG (Figure 1B—the M1 + 4 variant). The results (Figure 1C, red distribution) show that the fluorescence signal of M1 + 4 is greater than that of M2 but smaller than that of M1. To test whether this increase in fluorescence was due to spontaneous frameshifting from the M1 + 4 into the original M1 frame before the TAA stop codon, at either the transcriptional or the translational level, we extracted and sequenced the mRNA as well as carried out proteomic mass-spectrometry analysis on purified protein products from the M1 and M1 + 4 variants. The proteomics results (Figure S1A,B) show that proteins extracted from the M1 + 4 variant align almost exclusively with what is expected for the M2 variant, thereby excluding the option of spontaneous frame shifting back to the original M1 frame. In addition, sequence analysis of the mRNA (Figure S1C,D) shows that no deviation from the encoded sequence was observed for either the M1 or the M1 + 4 sequences. Finally, to further test the effects of an upstream nfORF and a resultant “nonsense” peptide encoding sequence, we mutated the M1 + 4 sequence to create four more HESP variants (Figure 1B, bottom): two lacking a start codon and two lacking a stop codon. The data show (Figure 1D) that all four variants exhibited a significant decline in mCherry fluorescence as compared with M1 + 4. Two of the four variants exhibited no fluorescence at all, while the other two exhibited a residual fluorescence that was similar to, or slightly less than, the fluorescence of the M2 variant. Consequently, it would seem that any boosting HESP nfORF sequence would need to have a proper translation frame (i.e., containing both start and stop codons).

To further characterize the sequences that lead to translation boost within a HESP framework, we constructed 35 additional variants that were divided into three groups: variants that varied the HESP length by adding one or more AAC codons (Figure 2A, left), variants that increased the distance between the HESP stop codon and the AUG of the tORF (Figure 2B, top-left), and variants that altered the distance between the internal HESP SD sequence and the HESP stop codon (Figure 2B, bottom-left). Finally, we mutated the internal M1 + 4 HESP SD sequence to variants with an increasing number of mismatches with respect to the 3′ tail of the ribosomal 16S rRNA.^17^ The data (Figure 2A, right) show that longer nfORFs generate an increasingly stronger fluorescent signal that seems to saturate with the insertion of the fourth codon and above. By contrast, increasing the distance between the stop codon and the tORF AUG has an inhibitory effect that culminates in a complete abolition of fluorescence once the inserted sequence reaches 12 nts. This is likely due to the increased energy barrier associated with ribosomal stretching^15^ from the internal nfORF SD to the tORF AUG, which ultimately becomes too large to overcome. Finally, the fluorescence mediated by the tORF (Figure 2C) shows a strong dependence on the number of matched bases of the nfORF SD with the 16S rRNA tail sequence, as expected.

**Figure 2:**
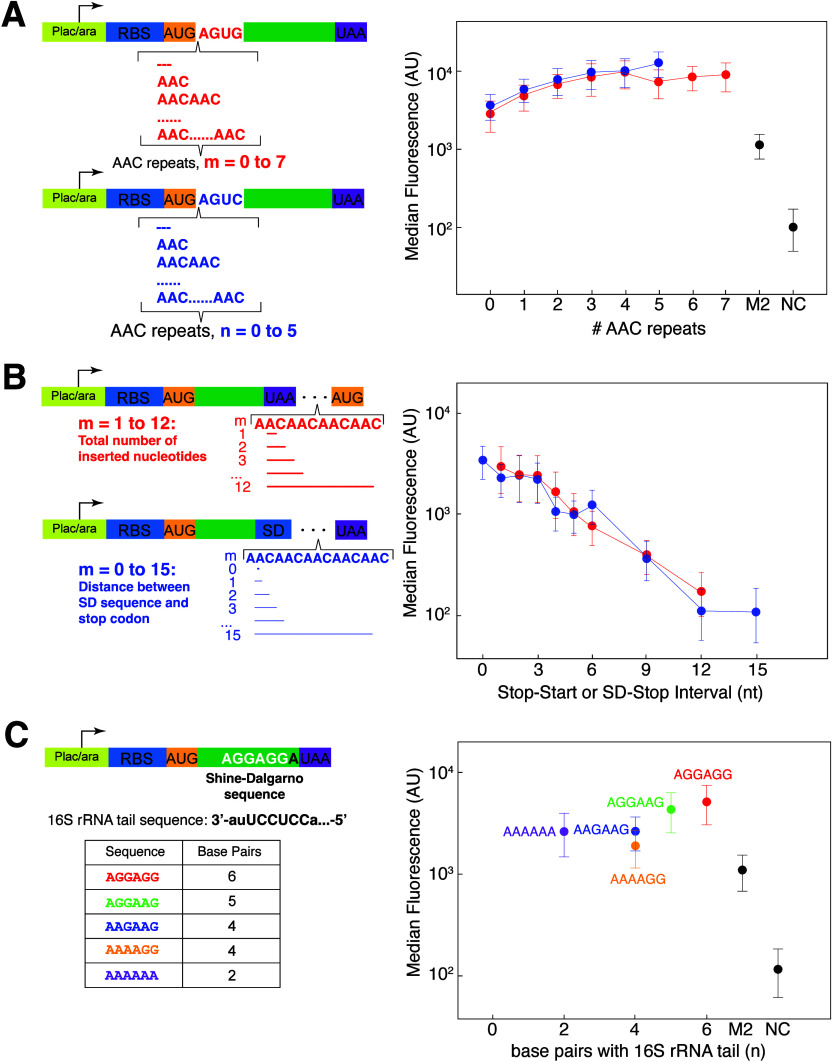
HESP boost dependent on several biophysical parameters. Exploration of HESP boost dependence on several biophysical parameters. For each panel the HESP sequences are shown on the left, while the median fluorescence is plotted on the right. For each variant, 2 technical repeats were carried out. Flow cytometry data from the Y2-A (615/20 nm filter, area) channel were extracted for each sample; the median fluorescence intensity was calculated as the main value for plotting. The error bars represent ±0.5 × IQR (interquartile range) to reflect data dispersion. Biophysical parameters: (A) HESP nfORF length (D1); (B) Distances between (red) HESP nfORF stop codon and tORF AUG (D2) and (blue) SD encoded within HESP nfORF and the HESP stop codon (D3); (C) HESP boost dependence on internal SD sequence.

To provide a more quantitative set of guidelines for HESP nfORF design, we developed a multilinear regression (MLR) model (Figure 3A) that was trained by the fluorescence data measured for 48 HESP variants (see Table S2 for variants and Table S3 for model parameters). We tested several regression models (see Figure S2 for alternative designs) and found that the best performing model necessitated 6 independent variables: the length of the short nfORF (D1), a summation of the distances between the HESP stop and tORF AUG (D2) and between the HESP SD sequence and its stop codon (D3), the number of base-pairs of the internal HESP SD with the 16S-rRNA tail (D4), and the RBS calculator predictions^17^ for the translational levels of the HESP nfORF (RBS) and tORF (RBS’). The 6-parameter model was able to generate an *R* (Pearson) of 0.8871 on this training data and outperformed all other model variations (Figure 3B, left and Figure S2). To validate the model’s prediction, we constructed three previously unseen variants of HESP nfORFs containing a 6xHis-tag codon insertion either upstream to or downstream to the internal HESP SD sequence (Figure 3B, right). The results, marked (1, 2, 3) on Figure 3B, left, show a strong agreement with the model’s prediction and do not deviate from the behavior of the training population.

**Figure 3:**
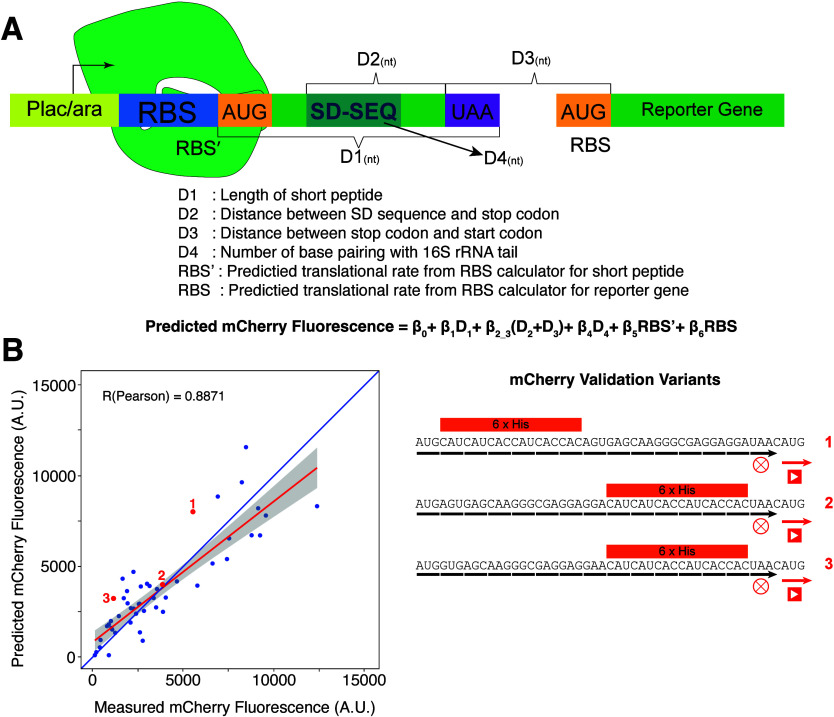
Model for HESP-generated translationally coupled boost. (A) Schematic model depicting all parameters involved in the regression model for the HESP boost (see equation). (B) (Left) Plot depicting measured vs predicted mCherry fluorescence generated by the HESP variants (model and measurements correlate with a Pearson correlation *R* of ∼0.88); (right) sequences for the M2 validation variants depicted in plot to the left (numbered red dots).

To provide a stronger validation for the regulatory role that the HESP sfORF may play in translation, we replaced the M2 tORF with the gene encoding for EGFP. We first tested for a boost in expression by inserting the HESP sequence from the M1 + 4 variant HESP­(30,7,7,6) upstream of the EGFP gene, and two additional HESP variants that lack either a start or stop codon, respectively (Figure 4A, top—note the transition to the general notation of HESP­(D1,D2,D3,D4)). The data show an ∼5× boost in expression generated by the HESP­(30,7,7,6) variant as compared with the EGFP ORF that lacks the HESP nfORF. Interestingly, both the no-start and no-stop variants also generated a slight boost in this case, albeit reduced as compared with the nonmutated HESP­(30,7,7,6), suggesting that ribosomal-induced unfolding of mRNA and translation coupling can occur in more challenging sequence settings. Finally, we tested the boost effect on EGFP translation for 6 additional HESP variants and found increases in expression from ×7 to ∼×20 (Figure 4B). These boosts correlated strongly with the boosts measured for the M2 variants containing the same HESPs (Figure S3), indicating that our predictive HESP boost model is relevant not only to the M2 variant but for other target ORFs as well.

**Figure 4:**
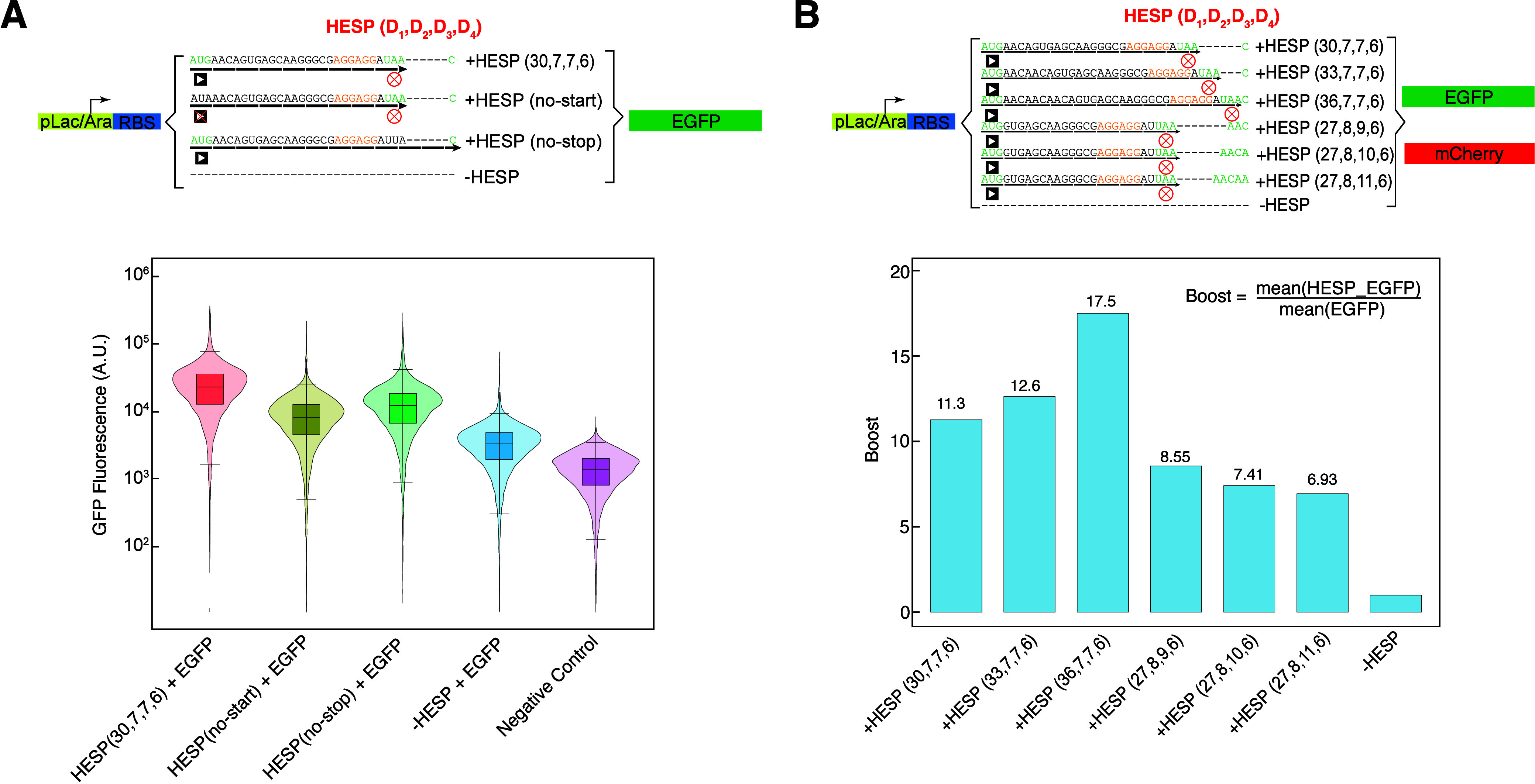
Validation of HESP-boost model with EGFP target ORF (A). Flow cytometry distributions of 4 EGFP HESP variants, depicted as violin plots. Sequence schematics are depicted at the top. (B) Fluorescence boost computed as shown in the inset for 6 additional HESP variants whose sequences are depicted above the panel.

## Discussion

We demonstrated that a short coding sequence positioned immediately upstream of a target gene can lead to a substantial boost in translation levels in bacteria, provided that the nonsense sequence contains a strong ribosome binding site 4 nucleotides upstream of the target gene AUG, and that the length of the nonsense peptide ORF is not smaller than 8–10 codons. This result suggests that the up-regulatory effect is caused by a fully assembled and translating ribosome that is capable of unfolding any adjacent mRNA structure, thus facilitating easier access to the ribosome binding site of the downstream gene, either for itself, namely, reinitiation, or for another ribosome, namely, translational coupling. This mechanism takes advantage of the fact that typically mRNA is known^18,19^ to be structured in the 5′ UTR and initiation regions of genes, and thus positioning another short translating region immediately upstream can lead to immediate boost in translation by this steric unwinding mechanism.

To predict the optimal nfORF sequence, we developed a regression model that takes into account a number of fundamental physical parameters (nfORF length, target gene separation, etc.), the sequence of the target gene, and previous thermodynamic model predictions that account for the translation initiation in the upstream RBS and reinitiation of the downstream RBS. Our model is therefore an expanded version of a model introduced by Tian and Salis^15^ in 2015, where they demonstrated a mechanistic framework that quantitatively predicted how multiple genes on a single polycistronic mRNA could be coordinated through translational coupling. Specifically, our model takes into account the length of the nfORF or short peptide mRNA coding sequence, whose effect on expression can be quantified by the probability for having either one SD or both SDs occupied by an initiating ribosome. This probability is dependent on the “protected” region or ribosomal footprint (∼10 nt upstream of the SD and ∼13–15 downstream of the AUG). Therefore, the longer the HESP coding region, the more likely the double occupancy scenario is to occur (Figure 2A) until complete deprotection of both ORFs is reached. Given the complexity of the mechanistic description, we opted to express this model via a regression approach to provide a more user-friendly prediction tool that can be implemented easily by anyone attempting to add the HESP peptide coding sequence into their expression system.

The nfORF based translational boosting reported here joins another result reported recently from our lab, where boosting was achieved by reducing the localized degradation of mRNA that is stored within phase-separated biocondensates.^20^ Together, these two technologies can be combined to engineer “hyper-expressing” bacterial cells. By hyperexpression, we mean expression levels that go beyond the overexpression state-of-the-art. However, we caution that unchecked utilization of expression boosting mechanisms may eventually lead to various toxicity and growth lagging effects that will ultimately defeat the purpose of these tools. To properly implement hyperexpression, an optimization tool that can properly predict the maximal possible expression level that can be generated for any given target gene and tolerated by the cell needs to be developed. Such optimized hyperexpression cells may substantially increase the titer of many industrially relevant proteins that are currently still too expensive to manufacture within a fermentation facility as compared with naturally sourcing them. As a result, such tools will likely open new opportunities for industry from the biotechnology, food, and pharmaceutical sectors.

## Supplementary Material





## Data Availability

All variant sequences, as well as additional data plotted in the figures in this article, are available in the Supporting Information.

## Notes

(Table S1) all HESP variants; (Table S2) HESP variants used for model calculations in Figure 3; (Table S3) MLR model parameter values (XLSX)
(Figure S1) Mass spectrometry and RNA analysis; (Figure S2) HESP effect MLR-based modeling; (Figure S3) EGFP and mCherry reporter levels correlation (DOC)
