# Supplementary material for: Boosting Gene Translation by a Short ORF Encoding for a “Nonsense” Peptide Positioned Immediately Upstream
Source: ACS Synth Biol. 2025 Oct 27;14(11):4314–21. doi: 10.1021/acssynbio.5c00505 (PMC13255528; doi:10.1021/acssynbio.5c00505)
Supplement: Supplementary file 2 [file sb5c00505_si_002.docx]

**Supporting Information for**

**Boosting gene translation by a short ORF encoding for a** “**nonsense**” **peptide positioned immediately upstream**

**Authors**: Junyi Cao^1^, Sarah Goldberg^1^ and Roee Amit^1,2*^

**Affiliations**:

1 Department of Biotechnology and Food Engineering, Technion - Israel Institute of Technology, Haifa 32000, Israel.

2 The Russell Berrie Nanotechnology Institute, Technion - Israel Institute of Technology, Haifa 32000, Israel.

* To whom correspondence should be addressed: [roeeamit@technion.ac.il](mailto:roeeamit@technion.ac.il).

**Figure S1: Mass spectrometry and RNA analysis exclude ribosomal or RNA frame-shifting**. (A) Amino acid abundance as a function of position for the M1 (top, right pellet) and M1+4 (bottom, left pellet) variants. In both cases the mCherry protein was purified via C-terminal His-tag and analyzed using MS/MS. MS raw data are supplied as Supplementary data files. (B) Abundance ratio between the M1 and M1+4 variants as a function of position showing that M1+4 exhibits essentially no signature for the first 10 amino acids, which is consistent with the existence of separate reading frame for the nonsense peptide. (C) Schematic for cDNA sequencing pipeline on a nanopore. (D) Table showing that cDNA from both M1 and M1+4 yielded predominantly the expected sequence without any evidence for a significant transcriptional shift error.

**Figure S2: MLR-based Modelling of HESP effect.** Panels representing different regression models for all 48 HESP variants used for model-building (see supplementary file for model variants). The top of each panel displays the Pearson correlation between experimental data and model prediction, as well as the model variables D1 - nfORF length, D2 - distance between HESP stop and mCherry AUG (M2), D3 - between HESP SD and HESP stop, RBS - RBS calculator prediction for HESP RBS, RBS' - RBS calculator prediction for mCherry RBS. All values were normalized by subtracting the negative control, making the negative control equivalent to zero in this plot. Thus, all reported median values represent differences relative to the negative control. Full model is depicted in Figure 3.

**Figure S3: Correlation between EGFP and mCherry reporter levels.** EGFP levels correlate strongly with mCherry levels for the same HESP variants. This supports the notion that the model can predict optimal HESP variants for any target gene. HESP nomenclature is as follows: HESP(D1,D2,D3,D4). Data points represent the mean of flow cytometry results for each of the HESP variants. Error bars represent the standard deviation for each variant from 10 technical repeat measurements.
